# Long-Term Zidovudine Therapy and Whether It is a Trigger of Vitamin B12 Deficiency: A Case Study of Megaloblastic Anemia at the University of Zambia Teaching Hospital

**DOI:** 10.1155/2022/3827616

**Published:** 2022-10-07

**Authors:** Natasha Mupeta Kaweme, Sahar Mounir Nagib Butress, Hamakwa Muluti Mantina

**Affiliations:** University Teaching Hospital, Private Bag RW1X Ridgeway, Nationalist Road, Lusaka 10101, Zambia

## Abstract

Macrocytic anemia is frequently observed in adult HIV-infected patients treated with nucleoside reverse transcriptase inhibitors and with vitamin B12 and folate deficiency. In this case report, we discuss a 52-year-old nonvegetarian male on long-term antiretroviral therapy for 5 years, presenting with severe macrocytic anemia (hemoglobin, 3.7 g/dL; mean corpuscular volume, 119.6 fL) and leukopenia (2.71*∗*10^9^/L), who was diagnosed with megaloblastic anemia caused by vitamin B12 deficiency following laboratory investigations. Parenteral vitamin B12 replacement therapy was initiated, with an early response observed. Notwithstanding, the treatment response was not sustained as the patient later presented with refractory anemia and persistence of macrocytosis. Discontinuation of zidovudine with concurrent vitamin B12 administration promptly improved the patient's clinical deficiency symptoms. At the end of 3 months, the patient had a complete hematological recovery. The deficiency of vitamin B12 disrupts DNA synthesis inhibiting effective hematopoiesis in all cell lines, particularly erythroid precursors and further promotes reversible bone marrow failure. Long-term ART therapy with zidovudine causes cytotoxicity in myeloid and erythroid precursors and induces bone marrow suppression. Whether long-term zidovudine consumption induced lower levels of vitamin B12 and subsequent megaloblastic anemia requires in-depth research and exploration.

## 1. Introduction

Anemia is a frequent hematological complication of human immunodeficiency virus (HIV) infection, and its incidence is adversely associated with advanced HIV disease, poor treatment outcomes, and increased risk of patient mortality [1, 2]. Although the causes of anemia in the general population are multifactorial in origin with various pathophysiological mechanisms, the most common etiology globally is iron deficiency, among other nutritional deficiencies such as folic acid and vitamin B12 [1]. In the setting of HIV infection, several factors, including chronic disease, the direct/indirect influence of HIV infection on hematopoietic stem/progenitor cells [3], altered cytokine milieu, opportunistic infections, and ART (antiretroviral therapy)-induced toxicity, result in impaired hematopoiesis and suppressed proliferation and differentiation of hematopoietic stem/progenitor cells, thus leading to cytopenias, anemia, thrombocytopenia, and neutropenia [4–6].

Although the burden of anemia among persons living with HIV in Zambia has not been fully established, a recent study by Cao et al. reported that the prevalence of anemia among adults living with HIV was higher in Southern Africa than that in East Africa. Furthermore, anemia in HIV infection was common among all groups in the population [2] and was higher in advanced HIV disease, among women, infants, and children in developing countries [7, 8]. Other factors, including socioeconomic factors, marital status, monthly income, and educational status, influence the severity of anemia among persons living with HIV [9].

Macrocytic anemia, defined as an increase in red blood cell mean corpuscular volume (MCV) of greater than 100 femtoliters (fL) in the setting of anemia, is a key observation in patients on long-term nucleoside reverse transcriptase inhibitors (NRTIs) [10, 11]. The exact mechanism by which NRTIs such as zidovudine led to macrocytosis in HIV-infected persons is yet to be explored. NRTIs interfere with mitochondrial DNA synthesis by inhibiting polymerases responsible for erythrocyte formation. Interference with mitochondrial DNA synthesis impairs the synthesis of erythroid precursors in the bone marrow (megaloblastic erythropoiesis) and consequently, macrocytosis [12–14]. Similarly, Reddy et al. hypothesized that zidovudine suppressed erythropoiesis and inhibited erythroid stem cells resulting in red cell aplasia, decreased reticulocyte counts and hemoglobin levels without hemolysis or blood loss [15], and increased MCV and erythropoietin levels [16]. A study conducted 3 decades ago highlighted that life-threatening hematological toxicity was probably attributed to transient depletion of mitochondrial DNA and sensitivity of DNA polymerase in some cell mitochondria [17].

At present, zidovudine and stavudine are less commonly used, as novel ART combinations with safer toxicity profiles have been introduced. In pediatric patients, zidovudine has been extensively used for treating HIV and preventing perinatal transmission of HIV in neonates [18]. However, the adverse mitochondrial toxicity of zidovudine favors the use of abacavir or tenofovir alafenamide. Zidovudine-induced bone marrow toxicity is common in patients with advanced disease and is related to early long-term high-dose therapy [19]. Generally, stavudine is associated with significant toxicity and increases the risk of macrocytosis, limiting its use in clinical practice [10].

According to the Zambia Consolidated HIV guidelines 2020 (currently in use), zidovudine-based regimens are the preferred second-line regimen for patients failing on tenofovir or abacavir-based regimens. A major concern for this arises from a recent study in Zambia that demonstrated high prevalence of HIV drug resistance, including thymidine analog mutations that confer resistance or reduced susceptibility to zidovudine, stavudine, and other NRTIs in adolescents and young adult patients [20].

This case study will discuss our experience with a patient who presented with macrocytic anemia associated with a history of long-term zidovudine consumption coupled with vitamin B12 deficiency and hypothesize the importance of a pragmatic approach to the diagnosis and management of macrocytic anemia in persons living with HIV to ensure the provision of quality health care in a resource-limited setting.

## 2. Case Presentation

A 52-year-old nonvegetarian male was referred to our hematology department with a one-month history of swelling of both lower limbs, progressive generalized body weakness, and severe anemia. He was diagnosed with HIV infection in 2010 and was on antiretroviral drugs for approximately 11 years at the time of presentation. The patient reported that his initial regimen was tenofovir/emtricitabine/efavirenz. He had no prior history of alcohol consumption and reported no HIV-related opportunistic infections during his course of treatment. His physical examination was significant for severe palmar and conjunctival pallor.

Initial laboratory results showed bicytopenia, hemoglobin 3.7 g/dL (reference range, 14.3–18.3), white cell count 2.71*∗*10^9^/L (reference range, 4.00–10.00), and a normal platelet count of 301*∗*10^9^/L (reference range, 150–400) with an elevated mean corpuscular volume (MCV) and mean cell hemoglobin (MCH) of 119.6 femtoliters (fL) (reference range, 79.1–98.9) and 40.2 picograms (pg) (reference range, 27.0–32.0), respectively. Peripheral blood differential counts were 55.3% neutrophils, 33.6% lymphocytes, 10.7% monocytes, 0.4% eosinophils, and no basophils. The peripheral blood smear showed anisopoikilocytosis, ovalomacrocytosis, and hypersegmented neutrophils. Serum vitamin B12 was 66.2 pg/ml (reference range, 211–946), and serum folate was 7.75 ng/ml (reference range 4.6–34).

A comprehensive metabolic panel, including kidney function tests, liver function tests, and a lipid panel, was within the normal range. Additional investigations such as stool for occult blood and echocardiogram/electrocardiogram showed no abnormalities. The patient had a complete viral suppression (target not detected) with an absolute CD4 count of 451 cells/*µ*L (reference range, 410–1590).The laboratory test results indicating a significant deficiency of vitamin B12 (66.2 pg/ml) and peripheral blood smear findings of hypersegmented neutrophils and oval macrocytes led the team to the diagnosis of megaloblastic anemia.

The patient received 4 units of packed cells while admitted, and parenteral vitamin B12 at 1000 mcg daily was initiated. At subsequent clinic visits, recovery of blood cell counts with normalization of MCV and MCH parameters was noted. The serum intrinsic factor antibody test result was equivocal. Despite this, maintenance of vitamin B12 at weekly and then monthly dosing was continued.

Two months after initiation of vitamin B12, the patient presented to our outpatient clinic with a complaint of generalized body weakness and heart palpitations with severe conjunctival pallor and tachycardia on examination. A complete blood count showed severe anemia with hemoglobin 3.6 g/dl, white cell count 2.41*∗*10^9^/l, MCV 114.1 fL, MCH 36.4 pg, and a normal platelet count. Peripheral blood smear findings were consistent with the initial report but showed significant numbers of round macrocytes, occasional oval macrocytes, and hypersegmented neutrophils. The patient's laboratory workup is summarized in Table 1.

After a detailed review of the patient's drug history, it was found that his antiretroviral medication was changed to an AZT-based regimen (zidovudine/lamivudine/efavirenz) in 2017 for unknown reasons. We decided to admit the patient for packed cell transfusion, and a consult was sent to the infectious disease unit to revise the treatment regimen. Following transfusion, the patient was discharged on vitamin B12, folic acid, and a dolutegravir-based regimen (tenofovir disoproxil fumarate/lamivudine/dolutegravir) based on the current WHO recommendations for first-line and second-line antiretroviral regimens. The patient was closely followed up in the outpatient department and experienced complete recovery of hemoglobin, red cell count, MCV, and MCH count with an improving absolute neutrophil count within 3 months. At present, the patient reports good physical health with stable blood counts.

## 3. Discussion

Macrocytic anemia is broadly classified as [1] megaloblastic associated with vitamin B12 and folate deficiency and [2] the nonmegaloblastic moiety associated with chronic alcoholism, liver disease, hypothyroidism, hereditary spherocytosis, and states of increased red cell consumption such as hemolysis or high turnover in pregnancy [21]. The most common cause of megaloblastic anemia is vitamin B12 deficiency caused by malabsorption due to the absence of intrinsic factors caused by pernicious anemia or following gastric surgery, transcobalamin II deficiency, duodenal and colonic inflammatory damage from celiac disease, or infection with the tapeworm *Diphyllobothrium latum*, and insufficient dietary intake. Common drugs such as phenytoin, trimethoprim, valproic acid, hydroxyurea, methotrexate, azathioprine, zidovudine, and other antiretroviral drugs can also cause macrocytic anemia [22].

Early identification and prompt treatment of vitamin B12 deficiency, a reversible and treatable cause of ineffective hematopoiesis, can significantly improve the survival outcomes of persons living with HIV. Our patient experienced refractory anemia and mild leukopenia despite continued treatment with parenteral vitamin B12 with no concern for other nutritional factors, opportunistic infections, or progression of HIV disease. At the initial diagnosis and confirmation of vitamin B12 deficiency and megaloblastic anemia, the patient showed an early response to vitamin B12 supplements; however, this early response was not sustained. We hypothesized that the relapse of megaloblastic anemia was probably associated with significantly low vitamin B12 levels, higher MCV levels, and the persistence of mitochondrial toxicity or the presence of mitochondrial DNA mutations induced by zidovudine which might elicit more severe mitochondrial dysfunction [21].

Although the patient's folate level of 7.75 ng/ml (reference range 4.6–34) was within the normal range (lower limit of normal), concern for ‘masked' folate deficiency after initiation of vitamin B12 was speculated. Vitamin B12 deficient cells are unable to utilize folate, and only upon treatment with vitamin B12, folate can be utilized. As a result, underlying folate deficiency can manifest with a significant decrease in folate levels of less than 2 ng/ml. Concurrent administration of folate with vitamin B12 will avert a suboptimal response of anemia to vitamin B12 alone. Measurement of red cell folate assay, serum methylmalonic acid, and homocysteine levels could have confirmed the likelihood of folate deficiency or combined nutritional deficiency. However, the tests were not performed due to financial constraints. In vitamin B12 deficiency, both metabolites increase proportionally to the severity of deficiency, whereas in pure folate deficiency, only homocysteine levels are elevated and methylmalonic acid is normal [23].

Desk review has not revealed any study that has explored the possibility of an association between zidovudine therapy and lower levels of vitamin B12 and folate and its impact on hematopoiesis and erythropoiesis. Notwithstanding, Puspasari et al. hypothesized that interference of mitochondrial DNA synthesis induced by zidovudine resulted in hematological interference in the form of reduced vitamin B12 and folate levels in blood which in turn, led to megaloblastic anemia and oral lesions [24, 25].

Cotrimoxazole prophylaxis is commonly used in Zambia for the prevention of pneumocystis jirovecii pneumonia, isosporiasis, toxoplasmosis, malaria, and other HIV-related and non-HIV-related diseases. It is initiated in adult patients with a CD4 count of less than 350 cells/*µ*L or having stage II, III, or IV disease according to the WHO clinical staging and discontinued when the CD4 count is greater than or equal to 350 cells/*µ*L for two consecutive values at least 6 months apart while on ART (Zambia Consolidated Guidelines for Treatment and Prevention of HIV Infection, 2020). Trimethoprim is considered a weak inhibitor of dihydrofolate reductase that can interfere with folic acid metabolism and promote megaloblastic changes at high doses [7]. In this case study, the patient reported no history of cotrimoxazole consumption; therefore, it was excluded as a possible cause of megaloblastic anemia.

Neutropenia is a common concomitant feature in megaloblastic anemia. Vitamin B12 and folate deficiency interferes with effective hematopoiesis and induces bone marrow suppression [26], decreased myeloid production, and adverse neutropenia which increases the risk of infection. Other etiologies, including bacterial and viral infections, immunodeficiency and underlying chronic disease, are commonly associated with leukopenia and neutropenia. In patients with infection due to neutropenia, underlying vitamin B12 deficiency may exacerbate the susceptibility to cytopenias [27].

Our patient had chronic but asymptomatic neutropenia, persisting for over three months. Notwithstanding, a gradual improvement in the total white cell count was observed with vitamin B12 treatment and zidovudine discontinuation. We hypothesized that the presence of chronic neutropenia could have been a result of prolonged or advanced vitamin deficiency in underlying HIV infection, although the patient had complete viral suppression. About 4 decades ago, Kaplan and Basford hypothesized that megaloblastic anemia of vitamin B12 and folate deficiency was associated with morphological and quantitative abnormalities in developing leucocytes, resulting in reduced leucocyte numbers with nuclei hypersegmentation [28].

Appropriate treatment of vitamin B12 deficiency provides rapid resolution of clinical deficiency symptoms and prompt hematological recovery. Our patient's biological markers reached normal values or improved within 3 months of zidovudine discontinuation and vitamin B12 supplementation. Furthermore, the patient had no clinical features consistent with deficiency. Initial response to vitamin B12 can be determined by reticulocytosis occurring within a week after starting treatment. Complete hematological recovery and normalization of MCV are estimated to be between 2 and 8 weeks [29]. The persistence of macrocytosis is not affected by the duration of zidovudine therapy. Yu et al. showed that a higher MCV took longer to normalize, with a percentage of patients having persistent macrocytosis 2 years after zidovudine cessation [21].

The treatment and follow-up of patients on antiretroviral drugs require a multidisciplinary approach, including ART specialists, hematologists, nutritionists, and pharmacologists, to ensure early identification of drug-induced macrocytosis and prompt intervention. Early nutritional intervention can ensure optimal nutrition and health status in persons receiving ART. Rezaei et al. suggested that combined administration of vitamin B12 and folate supplements had a beneficial effect on the hematological status of HIV-infected persons receiving HAART [30]. An urgent revision of first-line and second-line therapies to include novel integrase inhibitors such as dolutegravir, also currently available in resource-limited settings, can reduce the incidence of zidovudine-related macrocytic anemia. Zidovudine is avoided in patients with low hemoglobin levels due to its additive effect on anemia and myelotoxicity. Monthly monitoring of hemoglobin levels after switching and re-initiating ART drugs for three months should be mandatory.

Among the significant limitations in the management of our patient were the inability to measure serum methylmalonic acid and homocysteine levels to increase the sensitivity and precision of the diagnosis of vitamin B12, folate, or combined deficiency. These metabolites are useful markers when the clinical picture is equivocal. Second, a reticulocyte count was not possible, given the unavailability of methylene blue stain. Reticulocytopenia suggests vitamin B12 deficiency in megaloblastic anemia. The seminal limitation was the inability to assess vitamin B12 and folate serum levels following treatment to monitor response. The recent literature has revealed that monitoring vitamin B12 is not necessary for patients receiving replacement therapy.

## 4. Conclusion

Macrocytic anemia is frequently observed in HIV patients on long-term nucleoside reverse transcriptase inhibitors such as zidovudine. In this case study, we have discussed the history, presentation, and laboratory workup of a patient with refractory anemia and elevated mean corpuscular volume and mean corpuscular hemoglobin on long-term zidovudine therapy coupled with vitamin B12 deficiency, thus supporting a diagnosis of megaloblastic anemia. It may be possible that increased zidovudine toxicity was exacerbated by vitamin B12 deficiency, as each is associated with bone marrow suppression. Discontinuation of zidovudine and treatment with parenteral vitamin B12 resulted in a rapid resolution of the patient's clinical deficiency symptoms.

It is evident that anemia is prevalent among people living with HIV infection, and therefore, more attention is required for a tailored, safer choice of ART regimens to minimize drug-related hematological toxicities. It is highly recommended that older treatment regimens be discarded in preference for safer novel regimens that have demonstrated improved survival outcomes while minimizing adverse events. Accordingly, we propose a detailed study that will explore the link between long-term zidovudine therapy and lower levels of vitamin B12 and folate.

## Figures and Tables

**Table 1:** Laboratory results at initial diagnosis and at follow-up visits.

Laboratory parameters	Reference range	Initial laboratory results	Laboratory results over time following parenteral B12 supplementation			
			Post transfusion and B12 initiation	2 months post B12 supplementation	21 days post AZT discontinuation	3 months post AZT discontinuation
Hemoglobin (g/dL)	14.3–18.3	3.7	8.6	3.6	10.4	14.9
Red cell count (^*∗*^10^12^/L)	4.50–5.50	0.92	2.50	0.99	3.22	5.13
White cell count (^*∗*^10^9^/L)	4.00–10.00	2.71	2.58	2.41	2.61	3.56
Platelet count (^*∗*^10^9^/L)	150–400	301	320	281	188	200
Differential count (%)						
Neutrophils	40–70%	55.3%	51.9%	45.7%	36.3%	27.1%
Lymphocytes	20–40%	33.6%	36.4%	40.2%	52.5%	59.0%
Monocytes	2–10%	10.7%	9.7%	13.7%	7.7%	9.6
Eosinophils	0.04–0.4%	0.4%	1.6%	0.4%	3.1%	3.7
Basophils	0.02–0.2%	0.0%	0.4%	0.0%	0.4%	0.6%
MCV (fL)	79.1–98.9	119.6	98.8	114.1	103.4	87.3
MCH (pg)	27.0–32.0	40.2	34.4	35.4	32.3	29.0
Absolute neutrophil count (^*∗*^10^9^/L)	2.00–7.00	1.50	1.34	1.10	0.95	0.96
Urea (mmol/L)	2.80–7.10	4.12	—	—	—	4.83
Creatinine (*µ*mol/L)	59.0–104.0	58.3	—	62.4	—	89.6
Total bilirubin (mmol/L)	2.0–21.0	32.3	—	—	—	6.2
ALT (U/L)	0.0–45.0	20.0	—	17.1	—	8.2
AST U/L)	0.0–35.0	24.2	—	—	—	31.4
Lactate dehydrogenase (IU/L)	135–247	176	—	—	—	—

## Data Availability

All data underlying the results are available in the article, and no additional source data are required.

## Abbreviations

ART: Antiretroviral therapy
DNA: Deoxyribonucleic acid
HAART: Highly active antiretroviral therapy
MCH: Mean corpuscular hemoglobin
MCV: Mean corpuscular volume
HIV: Human immunodeficiency virus
NRTIs: Nucleoside reverse transcriptase inhibitors.

## References

[1] Zerihun K. W., Bikis G. A., Muhammad E. A. (2019). Prevalence and associated factors of anemia among adult human immune deficiency virus positive patients on anti-retroviral therapy at Debre Tabor hospital, Northwest Ethiopia. *BMC Research Notes*.

[2] Cao G., Wang Y., Wu Y., Jing W., Liu J., Liu M. (2022). Prevalence of anemia among people living with HIV: a systematic review and meta-analysis. *eClinicalMedicine*.

[3] Tsukamoto T., Gorantla S., Rodrigues V. (2021). Editorial: direct and indirect interactions of HIV with host cells. *Frontiers in Cellular and Infection Microbiology*.

[4] Ageru T. A., Koyra M. M., Gidebo K. D., Abiso T. L. (2019). Anemia and its associated factors among adult people living with human immunodeficiency virus at Wolaita Sodo University teaching referral hospital. *PLoS One*.

[5] Durandt C., Potgieter J. C., Mellet J. (2019). HIV and haematopoiesis. *South African Medical Journal*.

[6] Marchionatti A., Parisi M. M. (2021). Anemia and thrombocytopenia in people living with HIV/AIDS: a narrative literature review. *International Health*.

[7] Berhane Y., Haile D., Tolessa T. (2020). Anemia in HIV/aids patients on antiretroviral treatment at ayder specialized hospital, mekele, Ethiopia: a case-control study. *Journal of Blood Medicine*.

[8] Abioye A. I., Andersen C. T., Sudfeld C. R., Fawzi W. W. (2020). Anemia, iron status, and HIV: a systematic review of the evidence. *Advances in Nutrition*.

[9] Gebremedhin K. B., Haye T. B. (2019). Factors associated with anemia among people living with HIV/AIDS taking ART in Ethiopia. *Advances in Hematology*.

[10] Genn é D., Sudre P., Anwar D., Saa ï C., Hirschel B. (2000). Causes of macrocytosis in HIV-infected patients not treated with zidovudine. *Journal of Infection*.

[11] Moore C. A., Adil A. (2021). *Macrocytic Anemia*.

[12] Pertiwi D., Suradi, Winarni T. I., Probandari A. N. (2020). The length of AZT consumption and homocysteine levels are protective factors of macrocytic anemia among adult HIV-infected patients: a case-control study in central java province, Indonesia. *Bangladesh Journal of Medical Science*.

[13] Romanelli F., Empey K., Pomeroy C. (2002). Macrocytosis as an indicator of medication (zidovudine) adherence in patients with HIV infection. *AIDS Patient Care and STDs*.

[14] Kufel W. D. (2016). Nucleoside reverse transcriptase inhibitor (NRTI) associated macrocytosis. *International Journal of Virology AIDS*.

[15] Reddy D. M. Y., Lokesh A., Sivaranjani V., Lakshmi S. V., Subbaiah M. V. (2017). Zidovudine induced hematological disorders and hyperpigmentation. *World Journal of Pharmacy and Pharmaceutical Sciences*.

[16] Prashant J., Amit S., Mumtaz S., Ashok S. (2016). Zidovudine-induced anemia in HIV/AIDS. *New Indian Journal of Pediatrics*.

[17] Yarchoan R., Weinhold K., Lyerly H. K. (1986). Administration of 3’-AZIDO-3’-DEOXYTHYMIDINE, an inhibitor of htlv-III/lav replication, to patients with aids or aids-related complex. *The Lancet*.

[18] Antiretroviral medication zidovudine (ZDV, retrovir) | NIH. https://clinicalinfo.hiv.gov/en/guidelines.

[19] Getaneh Z., Wale W., Chanie B. (2021). Magnitude and associated factors of anemia among AZT based HAART experienced adult HIV patients at University of Gondar comprehensive specialized referral hospital, Northwest, Ethiopia, 2019: a retrospective cohort study. *BMC Infectious Diseases*.

[20] Miti S., Handema R., Mulenga L. (2020). Prevalence and characteristics of HIV drug resistance among antiretroviral treatment (ART) experienced adolescents and young adults living with HIV in Ndola, Zambia. *PLoS One*.

[21] Yu I., Greenberg R. N., Crawford T. N., Thornton A. C., Myint T. (2017). Persistence of macrocytosis after discontinuation of zidovudine in HIV-infected patients. *Journal of the International Association of Physicians in AIDS Care*.

[22] Wu Y. H., Hwang M. J., Lee Y. P., Chiang C. P. (2020). Atrophic glossitis in pernicious anemia patients can be treated to normal in two weeks by intramuscular injection of vitamin B12. *Journal of Dental Science*.

[23] Stabler S. P., Antony A. C. (2019). *Megaloblastic Anemia*.

[24] Puspasari D., Herawati D. M. D., Sufiawati I. (2018). Association between serum B12 and folate levels and manifestations of oral lesions in HIV adult patients. *Malaysian Journal of Nutrition*.

[25] Shiboski C. H., Chen H., Secours R. (2015). High accuracy of common HIV-related oral disease diagnoses by non-oral health specialists in the AIDS clinical trial group. *PLoS One*.

[26] Rs D., Majmundar K. (2017). B12 deficiency as a cause of neutropenic sepsis. *Annals of Hematology and Oncology*.

[27] Sarbay H. (2019). Investigation of the incidence of vitamin B12 deficiency in leukopenia and neutropenia secondary to infection. *International Journal of Contemporary Pediatrics*.

[28] Kaplan S. S., Basford R. E. (1976). Effect of vitamin B12 and folic acid deficiencies on neutrophil function. *Blood*.

[29] Carmel R. (2008). How i treat cobalamin (vitamin B12) deficiency. *Blood*.

[30] Rezaei E., Sedigh Ebrahim-Saraie H., Heidari H. (2016). Impact of vitamin supplements on HAART related hematological abnormalities in HIV-infected patients. *Medical Journal of the Islamic Republic of Iran*.

